# Supplementary material for: Menstrual flow as a non-invasive source of endometrial organoids
Source: Commun Biol. 2021 Jun 17;4:651. doi: 10.1038/s42003-021-02194-y (PMC8211845; doi:10.1038/s42003-021-02194-y)
Supplement: Supplementary file 2 — Supplementary Information [file 42003_2021_2194_MOESM2_ESM.pdf]

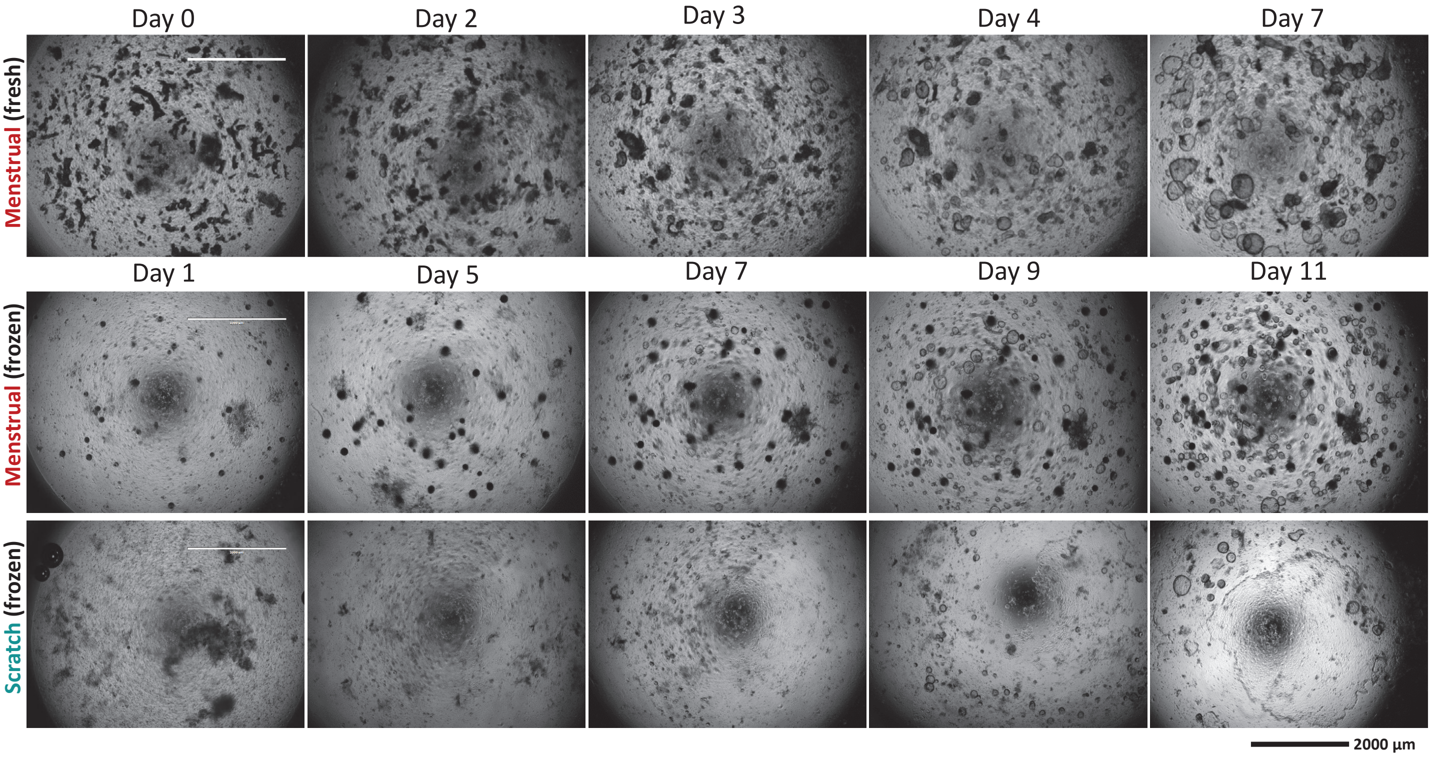

Supplementary Figure 1: **Derivation and characterisation of menstrual flow organoids**. Time line images detailing propagation of organoids from menstrual flow and scratch. The top panel represents the growth of menstrual organoids directly derived from menstrual blood. The middle and bottom panels illustrate the growth of organoids from frozen menstrual (middle) and scratch (bottom) digests of the same patient. Scale bar 2,000 µm.

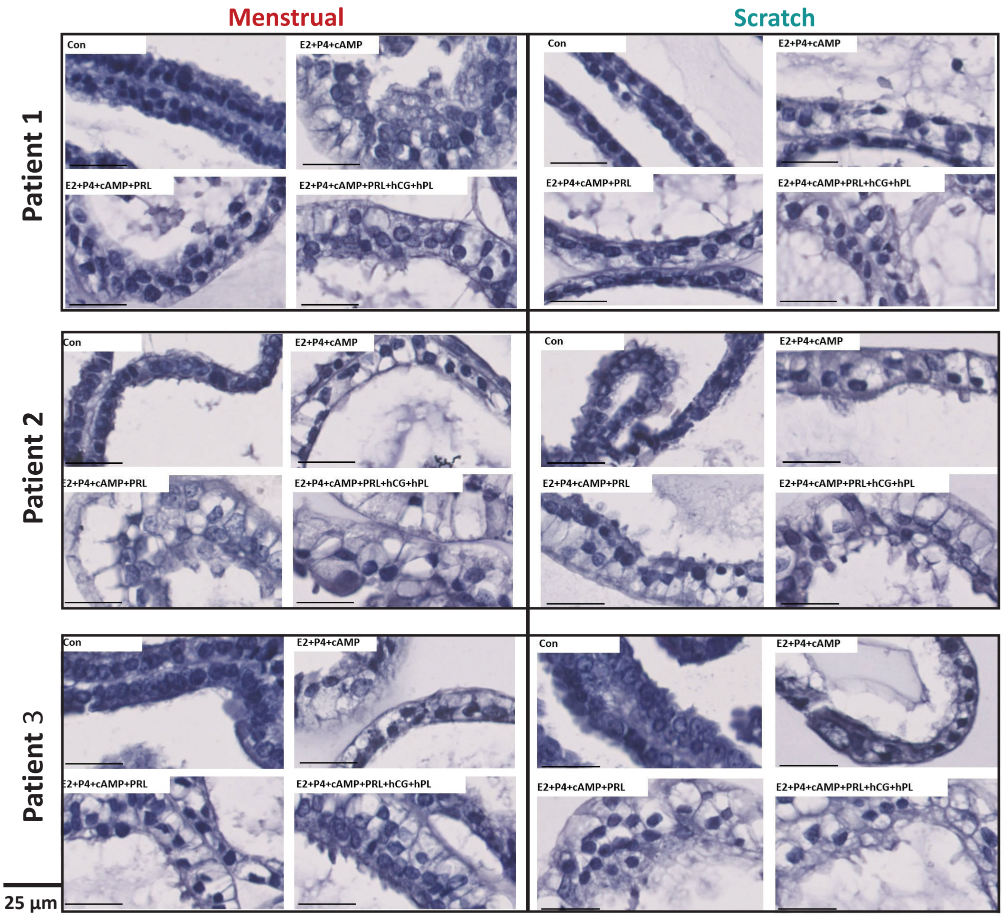

Supplementary Figure 2: Haematoxylin and eosin (H&E) staining of hormonally-treated menstrual and scratch organoids from three patients. Menstrual and scratch organoids were grown for 4 d, and treated with culture medium alone or β-estradiol for 2 d followed by β-estradiol, progesterone and cAMP (EPC), EPC plus prolactin, or EPC plus prolactin, hPL and hCG for 4 d. Paraffin-embedded sections were stained with H&E reagents. The images show morphological differences between untreated vs. hormonally treated organoids from 3 patients. There is evidence of a columnar epithelial morphology with increased vacuole formation analogous to the hypersecretory phenotype of early pregnancy. Scale bar 25 µm.

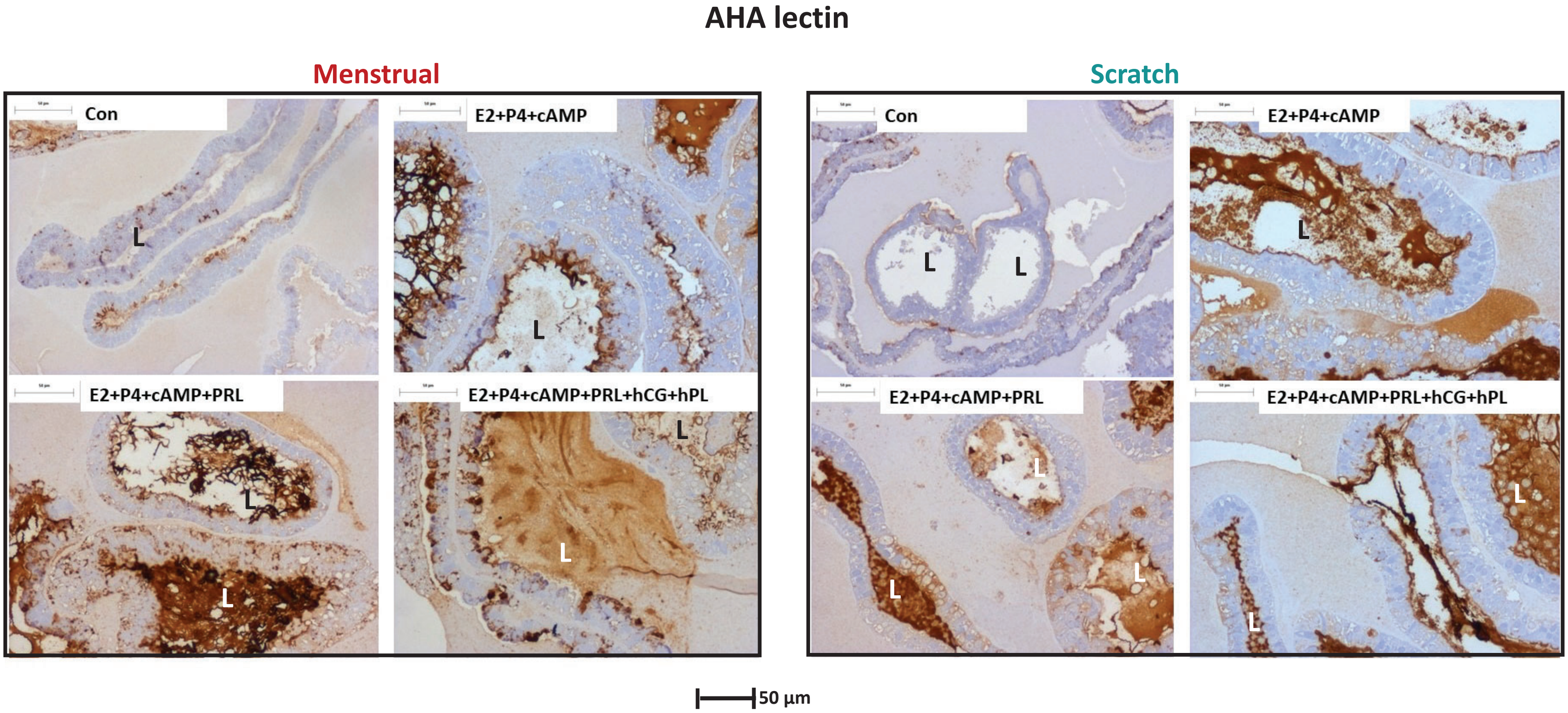

Supplementary Figure 3: ***Arachis hypogaea*** **agglutinin (AHA) lectin staining of semi-thin resin sections of menstrual and scratch organoids treated with hormones.** Menstrual and scratch organoids were grown for 4 d, and treated with culture medium alone or β-estradiol for 2 d followed by β-estradiol, progesterone and cAMP (EPC), EPC plus prolactin, or EPC plus prolactin, hPL and hCG for 4 d. Semi-thin resin sections were stained with AHA lectin. L = lumen; Scale bar 50 µm.

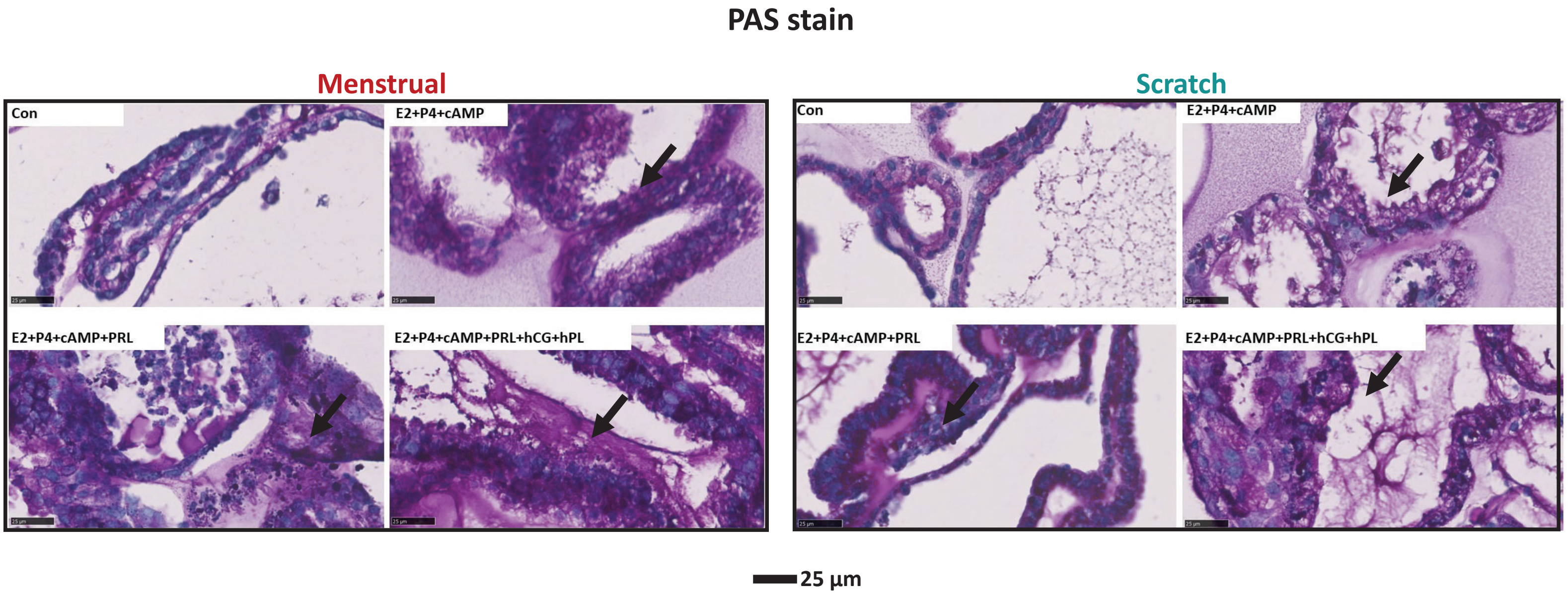

Supplementary Figure 4: **Periodic Acid Schiff (PAS) staining of menstrual and scratch organoids treated with hormones.** Menstrual and scratch organoids were grown for 4 d, and treated with culture medium alone or β-estradiol for 2 d followed by β-estradiol, progesterone and cAMP (EPC), EPC plus prolactin, or EPC plus prolactin, hPL and hCG for 4 d. Paraffin-embedded sections were stained with PAS reagents to detect glycogen (purple). Arrows indicate purple glycogen staining, which is also secreted into the lumen of organoids treated with hormones. Scale bar 25 µm.

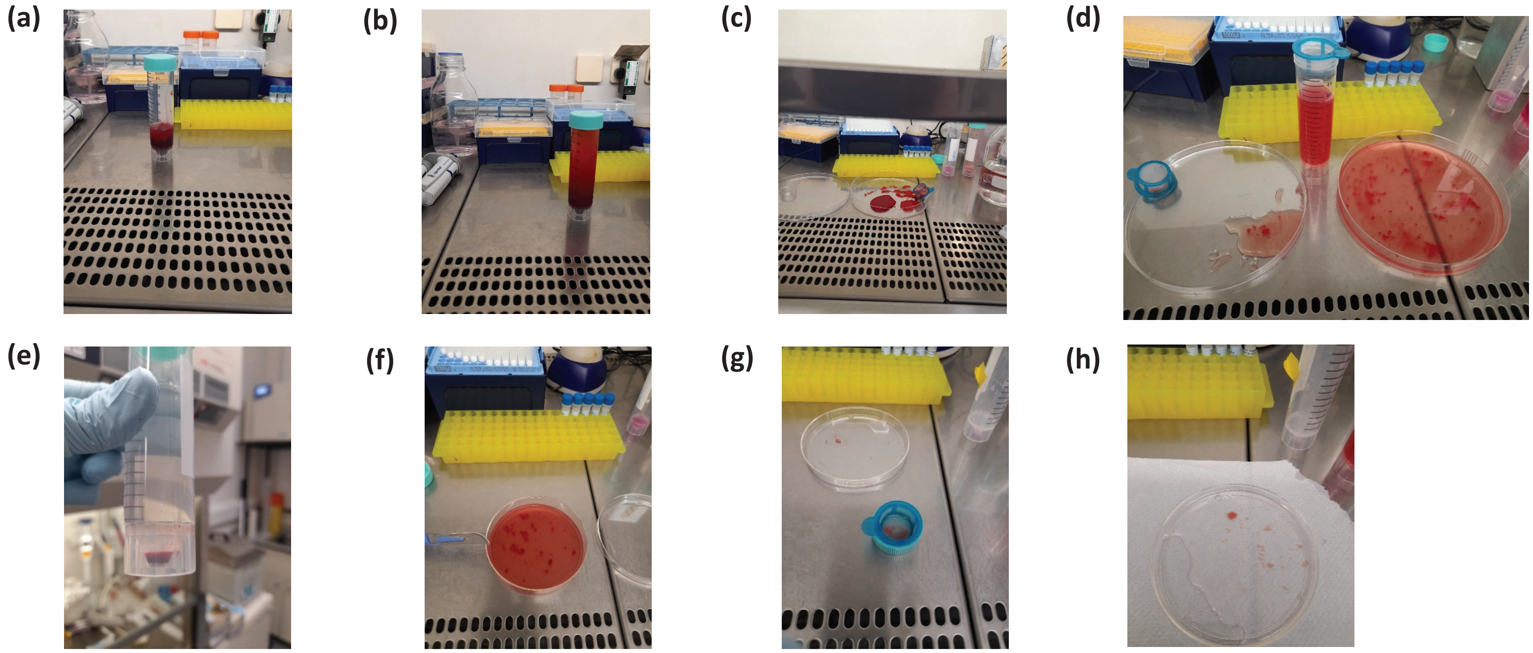

Supplementary Figure 5: Derivation of menstrual organoids. (a) Menstrual blood samples were spun down at 600 g for 5 min, and (b) washed with PBS several times to remove red blood cells, discarding the bloody supernatant and collecting only the cellular material after every wash. (c-d) The cellular sediment was subsequently passed through a 100-µm sieve (Corning, 431752) and washed with more PBS. The sieve was inverted over a Petri dish and retained cellular debris was backwashed from the sieve membranes, to collect the solid endometrial gland-containing material. (e) This resulted in eliminating most of the red blood cells. (f) Collected tissue debris was chopped with a scalpel into 5 mm fragments and digested in 20 ml 25 U ml^{-1} Dispase II (Sigma, D4693)/0.4 mg ml^{-1} collagenase V (Sigma, C-9263) solution in RPMI 1640 medium (Thermo Fisher Scientific, 21875-034)/10% FCS (Biosera, FB-1001) with gentle shaking at 37 °C for 20 min. (g) The solution was neutralised with 20 ml cold RPMI 1640/10% FBS medium and passed through a clean 100-µm sieve. h) The sieve was inverted again over a Petri dish and retained glandular elements were backwashed from sieve membranes, using vigorous bursts of medium from a disposable Pasteur pipette, pelleted by centrifugation and resuspended in ice cold Matrigel (Corning, 536231) at a volume: volume ratio of 1:20. 25-µl drops of Matrigel–cell suspension were plated into 48-well plates (Costar, 3548), allowed to set at 37 °C and overlaid with 250 µl organoid culture medium. The medium was changed every 2–3 d and cultures were passaged by manual pipetting every 7–10 d.

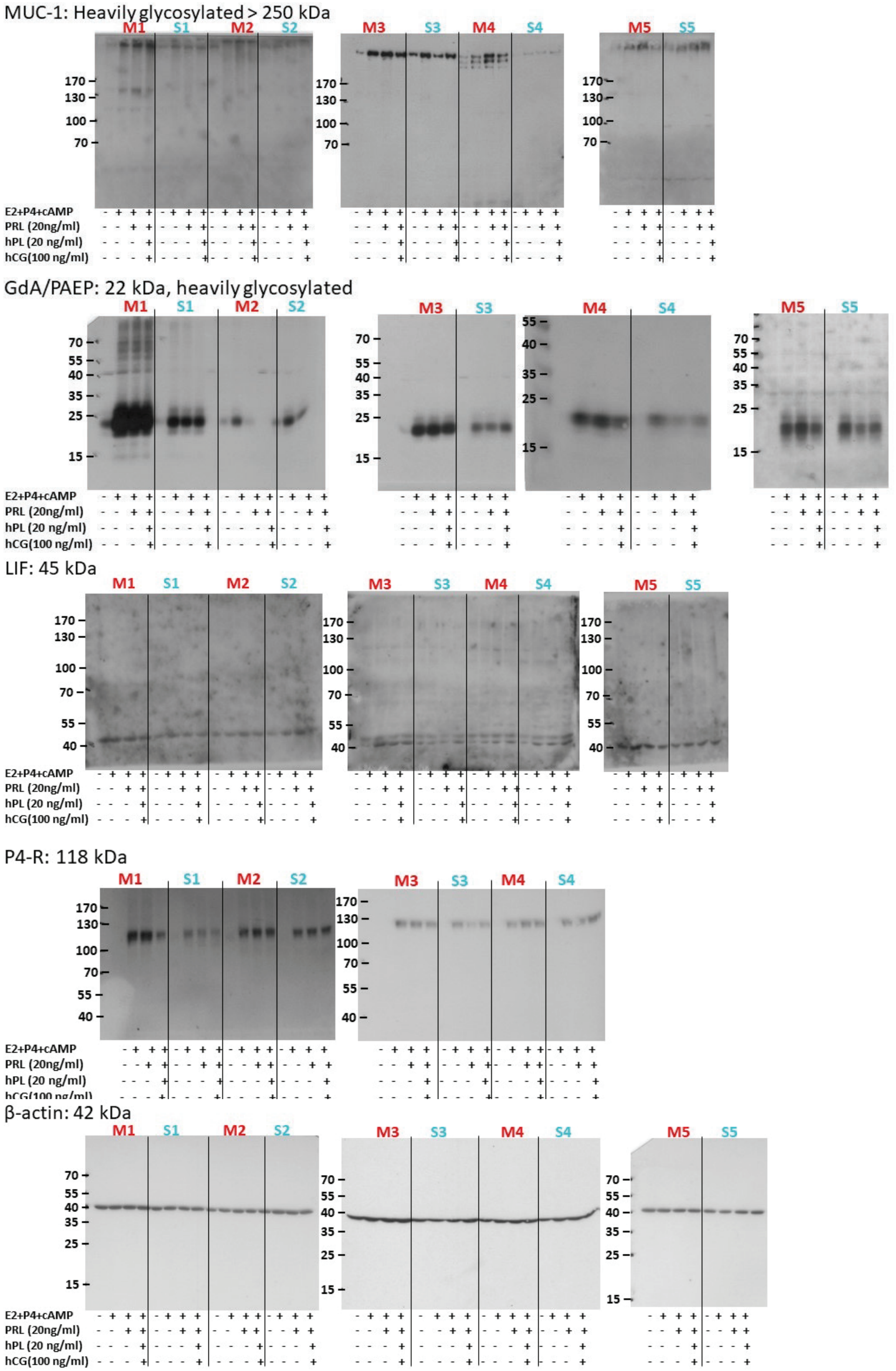

Supplementary Figure 6: Uncropped scans of western blots presented in Figs. 2f and 3b. All raw blots are presented. Western blots of menstrual (M) and scratch (S) samples from 5 patients were run and probed with anti-glycodelin (GdA/PAEP; Abcam, ab53289, 1:1000), anti-MUC-1 (Abcam, ab28081, 1:500), anti-LIF (Santa Cruz, sc-1336, 1:1000), anti-progesterone receptor (P4-R; Abcam, ab32085, 1:1000) or β-actin (Invitrogen, 15G5A11/E2) primary antibodies. These blots were used to quantify Figs. 2e and 3c. β-actin was used to normalise for gel loading. Molecular weights are indicated on the uncropped blots.

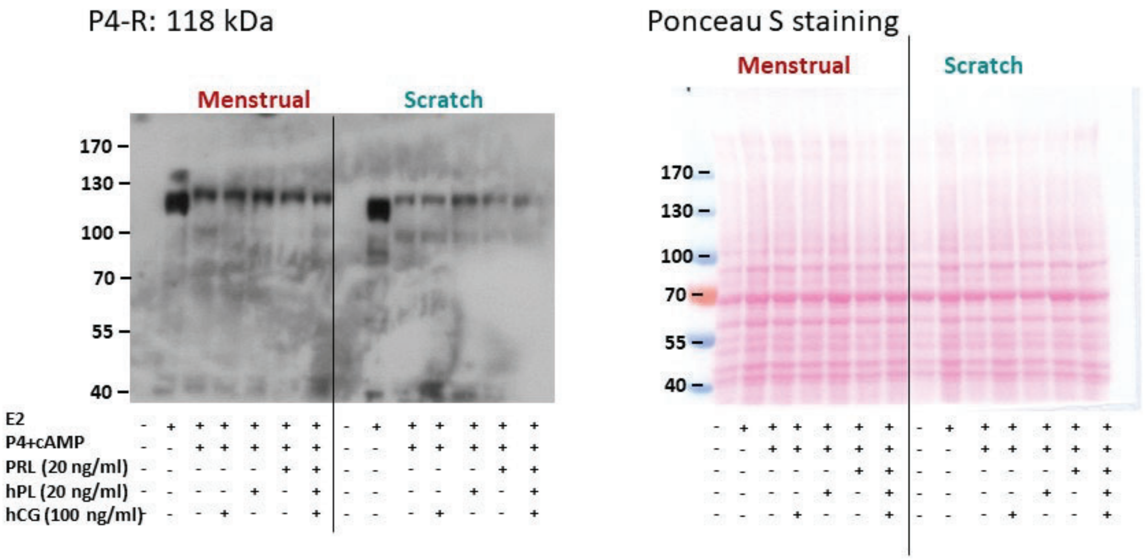

Supplementary Figure 7: Uncropped scans of western blots presented in Fig. 3d. Western blots of menstrual were run and probed with anti-progesterone receptor (P4-R; Abcam, ab32085, 1:1000) primary antibody. Molecular weights are indicated on the uncropped blots.
