# Supplementary material for: Menstrual flow as a non-invasive source of endometrial organoids
Source: Commun Biol. 2021 Jun 17;4:651. doi: 10.1038/s42003-021-02194-y (PMC8211845; doi:10.1038/s42003-021-02194-y)
Supplement: Supplementary file 3 — Reporting Summary [file 42003_2021_2194_MOESM3_ESM.pdf]

nature research

Corresponding author(s): Tereza Cindrova-Davies
COMMSBIO-20-2168A

Last updated by author(s): Apr 13, 2021

Reporting Summary

Nature Research wishes to improve the reproducibility of the work that we publish. This form provides structure for consistency and transparency in reporting. For further information on Nature Research policies, see our Editorial Policies and the Editorial Policy Checklist.

Statistics

For all statistical analyses, confirm that the following items are present in the figure legend, table legend, main text, or Methods section.

n/a	Confirmed	
☐	☒	The exact sample size (*n*) for each experimental group/condition, given as a discrete number and unit of measurement
☐	☒	A statement on whether measurements were taken from distinct samples or whether the same sample was measured repeatedly
☐	☒	The statistical test(s) used AND whether they are one- or two-sided<br>*Only common tests should be described solely by name; describe more complex techniques in the Methods section.*
☒	☐	A description of all covariates tested
☒	☐	A description of any assumptions or corrections, such as tests of normality and adjustment for multiple comparisons
☒	☐	A full description of the statistical parameters including central tendency (e.g. means) or other basic estimates (e.g. regression coefficient) AND variation (e.g. standard deviation) or associated estimates of uncertainty (e.g. confidence intervals)
☒	☐	For null hypothesis testing, the test statistic (e.g. F, t, r) with confidence intervals, effect sizes, degrees of freedom and P value noted<br>*Give P values as exact values whenever suitable.*
☒	☐	For Bayesian analysis, information on the choice of priors and Markov chain Monte Carlo settings
☒	☐	For hierarchical and complex designs, identification of the appropriate level for tests and full reporting of outcomes
☒	☐	Estimates of effect sizes (e.g. Cohen's *d*, Pearson's *r*), indicating how they were calculated

Our web collection on statistics for biologists contains articles on many of the points above.

Software and code

Policy information about availability of computer code

Data collection	Protein bands were quantified using Image J software (National Institutes of Health, http://rsb.info.nih.gov/ij/).
Data analysis	Original reads files were aligned to GRCh38 human genome (Ensembl Release 84) with STAR (v2.5.1b_modified)3. Alignments and quality control (QC) were processed using a ClusterFlow4 (v0.5dev, https://github.com/ewels/clusterflow) with the “fastqc_star” pipeline. QC reports were assessed using MultiQC5 (v0.9dev), which includes output from FastQC6 (v0.11.5), Trim_galore7 (v0.6.4) and fastq_screen8 (version 0.9.3). Gene quantification was determined with HTSeq-Counts9 (v0.6.1p1). Differential gene expression analysis was performed with DESeq2 (v1.26.0) package in R10 (v3.6.2).

For manuscripts utilizing custom algorithms or software that are central to the research but not yet described in published literature, software must be made available to editors and reviewers. We strongly encourage code deposition in a community repository (e.g. GitHub). See the Nature Research guidelines for submitting code & software for further information.

Data

Policy information about availability of data

All manuscripts must include a data availability statement. This statement should provide the following information, where applicable:

- Accession codes, unique identifiers, or web links for publicly available datasets
- A list of figures that have associated raw data
- A description of any restrictions on data availability

The RNA-Seq data sets have been deposited at:
http://www.ebi.ac.uk/arrayexpress/experiments/E-MTAB-9284
Figure 2b displays raw data.
All source data underlying the graphs and tables are available in Supplementary Data files and/or can be found in GitHub (https://github.com/CTR-BFX/Cindrova-

Davies_Turco) with corresponding DOI 10.5281/zenodo.4696302 (https://zenodo.org/record/4696302#.YHl2RGjTWjg). Microcopy images data are available in Cambridge research repository Apollo with DOI link (https://doi.org/10.17863/CAM.68319).

Field-specific reporting

Please select the one below that is the best fit for your research. If you are not sure, read the appropriate sections before making your selection.

- [x] Life sciences
- [ ] Behavioural & social sciences
- [ ] Ecological, evolutionary & environmental sciences

For a reference copy of the document with all sections, see nature.com/documents/nr-reporting-summary-flat.pdf

Life sciences study design

All studies must disclose on these points even when the disclosure is negative.

Sample size

This study utilised samples from two sets of patients. The first set consisted of healthy volunteers recruited for a pilot study, which was used only for collection of menstrual flow for set up of protocol. In this pilot study, we received samples of flow from 7 volunteers with normal cycles and succeeded in deriving organoids from 6 volunteers. A repeat sample from the volunteer whose culture was unsuccessful also failed. Cells isolated from the flow appeared dead, and we attributed this to sample collection/volunteer problems and did not explore the issue further. A further 3 volunteers provided repeat samples, and we successfully derived organoids from all.
The second set consisted of patient volunteers undergoing an IVF treatment cycle at the Bourn Hall Clinic, from whom we derived organoids from endometrial scratches and ensuing menstrual flow. This set of patients was used to validate and characterise the menstrual flow organoids. We received menstrual samples from 8 patients and succeeded in deriving organoids from 7. In the failed sample, the starting tissue appeared white and dead, but the cause was not explored further. Altogether, the success rate of deriving organoids from menstrual flow was 87%.

Data exclusions

No data were excluded

Replication

We were able to derive organoids from menstrual flow in 6/7 patients in the pilot study and 7/8 patient volunteers from the Bourn Hall Clinic. Altogether, the success rate of deriving organoids from menstrual flow was 87%.

Randomization

There were no experimental groups as the aim was to derive organoids for individual patients from two sources ' and endometrial scratch and subsequent menstrual flow. The pairs of organoids were then compared using RNA-Seq and stimulated with pregnancy hormones. There was no randomisation therefore.

Blinding

The aim was to compare organoids derived from two different sources but from the same patient. Blinding was therefore not appropriate.

Reporting for specific materials, systems and methods

We require information from authors about some types of materials, experimental systems and methods used in many studies. Here, indicate whether each material, system or method listed is relevant to your study. If you are not sure if a list item applies to your research, read the appropriate section before selecting a response.

Materials & experimental systems

n/a	Involved in the study	
☐	☒	Antibodies
☒	☐	Eukaryotic cell lines
☒	☐	Palaeontology and archaeology
☒	☐	Animals and other organisms
☐	☒	Human research participants
☒	☐	Clinical data
☒	☐	Dual use research of concern

Methods

n/a	Involved in the study	
☒	☐	ChIP-seq
☒	☐	Flow cytometry
☒	☐	MRI-based neuroimaging

Antibodies

Antibodies used

prolactin receptor (Preprotech, Cat no: 100-07), progesterone receptor (Dako, M3569), progesterone receptor (Abcam, ab32085), acetylated tubulin (Cell Signaling, #611B1), glycodelin (Abcam, ab53289), MUC-1 (Abcam, ab28081), LIF (Santa Cruz, sc-1336), Ki67 (Abnova, PAB12127)

Validation

prolactin receptor (Preprotech, Cat no: 100-07, for IHC used without antigen retrieval, 1:500), progesterone receptor (Dako, M3569, for IHC used with antigen retrieval (Tris-EDTA, pH 9), 1:50), progesterone receptor (Abcam, ab32085, for western blotting used at 1:1000), acetylated tubulin (Cell Signaling, #611B1, for IHC used with antigen retrieval (Tris-EDTA, pH 9), 1:1000), glycodelin (Abcam, ab53289, for IHC used with antigen retrieval (Tris-EDTA, pH 9) at 1:500; for western blotting used at 1:1000), MUC-1 (Abcam, ab28081, for western blotting used at 1:500), LIF (Santa Cruz, sc-1336, for western blotting used at 1:1000), Ki67 (Abnova,

PAB12127, for IHC used with antigen retrieval (Tris-EDTA, pH 9), 1:10,000)

Human research participants

Policy information about studies involving human research participants

Population characteristics	In the first pilot set of patient, we recruited 7 healthy volunteers for the pilot study with informed written consent (ethical approval HBREC.2017.10). Volunteers had normal cycles and included nulliparous and parous individuals. Samples were collected on an anonymised basis. Menstrual flow organoids were successfully derived from 6 of these samples. These pilot data were used only for collection of menstrual flow for set up of protocol as a proof of principle pilot design.<br>In the second set of patients, 7 patients who undertook elective endometrial scratch (ES) procedures prior to initiating a treatment cycle at Bourn Hall Clinic, Cambridge, also agreed to collect a sample of the menstrual flow that subsequently followed the ES procedure. Endometrial gland organoids were derived from a biopsy sample taken during the ES procedure, as well as from a sample of menstrual flow. These data were used to validate and characterise the menstrual flow organoids.
Recruitment	Pilot study healthy volunteers were recruited from the Department of Physiology, Development and Neuroscience. The volunteers were not asked any medical history, or personal information. The samples were anonymised, assigned a numerical code, and the identity of the volunteers was unknown to the researcher.<br>The second set of patients were recruited by the medical staff at Bourn Hall Clinic. Patients opting for an endometrial scratch as part of an IVF treatment cycle were invited to collect subsequent menstrual flow in an endometrial cup that was provided. Whether they agreed or not did not affect their clinical treatment.
Ethics oversight	The pilot study was approved by the University of Cambridge Human Biology Research Ethics Committee (ethical approval HBREC.2017.10).<br>All procedures in the Bourn Hall Clinic were carried out with informed written consent, and local ethics approval (East of England - Cambridge Central Research Ethics Committee 17/EE/0151).

Note that full information on the approval of the study protocol must also be provided in the manuscript.
